# The Battle Between Japanese Encephalitis Virus and Host Innate Immune System: Evasion and Response

**DOI:** 10.3390/v18090975

**Published:** 2026-09-04

**Authors:** Teng Liu, Shucheng Zong, Shengkui Xu, Huan Jin, Dengjin Chen, Zhenhua Zhang

**Affiliations:** 1 Institute of Animal Husbandry and Veterinary Medicine, Beijing Academy of Agricultural and Forestry Sciences, Beijing 100097, China; liuteng@baafs.net.cn (T.L.); jinhuan0717@126.com (H.J.); 2 China Animal Husbandry Industry Co., Ltd., Beijing 100095, China; zongshucheng6688@163.com; 3 Beijing Key Laboratory of Traditional Chinese Veterinary Medicine, College of Animal Science and Technology, Beijing University of Agriculture, 7 Beinong Road, Changping District, Beijing 102206, China; skxu0721@163.com

**Keywords:** Japanese encephalitis virus (JEV), innate immune responses, immune evasion strategies

## Abstract

Japanese encephalitis (JE) is a natural zoonotic disease caused by the Japanese encephalitis virus (JEV), which poses potential threats to human health and the pig farming industry. To establish infection, JEV must overcome the innate immune responses and complete its lifecycle in new hosts. Notably, the direct virus-induced neuronal cell death and an uncontrolled neuroinflammatory response jointly lead to the pathogenesis of JEV. In this review, we will focus on the innate immune response to JEV infection and the viral immune evasion strategies, such as escaping recognition or inhibiting the production of antiviral factors. Generally, JEV exploits four innate immune pathways, including type I interferon, interleukins, programmed cell death, and autophagy, to facilitate self-replication or exacerbate disease. Moreover, host microRNAs modulated during JEV infection have emerged as key regulators of this virus–host interplay. Therefore, a full understanding of how the immune system reacts to JEV infection and how the virus evades innate immune clearance will help develop effective vaccines or antiviral therapies.

## 1. Japanese Encephalitis Virus (JEV)

### 1.1. Epidemiology

Japanese encephalitis (JE) was reported in 1871 in Japan, and its causative agent, Japanese encephalitis virus (JEV), was first isolated in human brain tissue in 1935 [1]. In the following years, Korea (1933), China (1940), India (1955), and other Asian countries reported JE cases successively [2]. JEV is a leading cause of viral encephalitis and there are approximately 68,000 cases of JE annually and at least 13,000 deaths according to the World Health Organization (WHO) report [3,4]. Among these cases, the fatality rate for patients with encephalitis is approximately 30%, and approximately 30~50% of cases have permanent neurological or psychiatric sequelae, especially in children [3,5]. The clinical spectrum of JEV infection ranges from asymptomatic infection to fatal encephalitis. After an incubation period of 4–14 days, symptomatic patients typically develop a febrile prodrome characterized by headache, vomiting, and myalgia, which can progress to acute encephalitis with altered consciousness, seizures, and focal neurological deficits; a case-fatality rate of 20–30% and long-term neuropsychiatric sequelae in 30–50% of survivors remain major public health concerns [6].

As a dual-host virus, JEV is transmitted horizontally between arthropods and vertebrates [4]. Mosquitoes are recognized as the primary vectors and mainly transmit JEV to pigs, humans, and other animals, although JEV RNA has also been detected in host-questing tick nymphs in certain reports [7]; it remains an important zoonotic mosquito-borne orthoflavivirus. Pigs, the terminal host, usually develop high viral titers and prolonged viremia that facilitates virus transmission to mosquitoes [8]. Infection of pregnant sows can cause miscarriage or lead to mummified, weak, or stillborn piglets. In addition, humans and horses are considered to be terminal hosts because JEV infection in these species results in insufficient viremia to infect mosquitoes taking a blood meal [9]. Many factors such as the expanding global population, and climate fluctuations have contributed to the increased prevalence of JEV. To date, almost 2 billion people in endemic countries face the constant threat of JEV. To reduce the impact of JEV infection, a variety of vaccines, including live-attenuated, inactivated, and chimeric vaccines, have been applied in many countries and regions [10]. Although vaccination has contributed substantially to controlling JEV infection worldwide, the infection risks remain high in some developing countries and areas. The persistently high infection risk despite successful vaccination can be attributed to incomplete vaccine coverage in some regions, the presence of amplifying hosts such as pigs, and climate-driven changes in mosquito vector distribution [6].

### 1.2. The Virion

**Figure 1:**
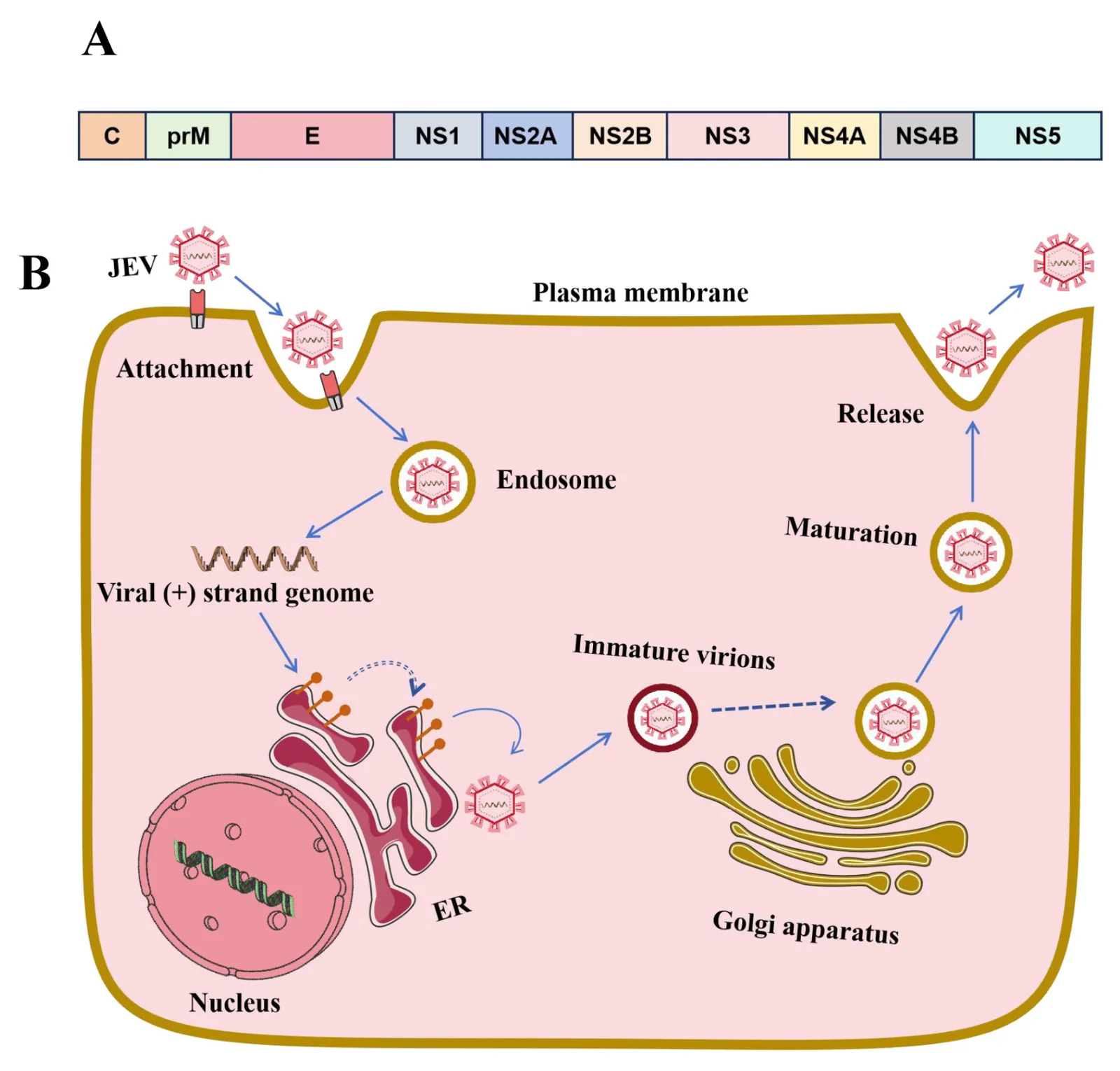
The genetic composition and replication cycle of JEV. (**A**). JEV positive-sense single-stranded RNA genome consists of 11,000 nucleotides and encodes three structural proteins, including the enveloped protein (E), the membrane protein (M), and the capsid protein (C), as well as seven non-structural proteins named NS1, NS2A/2B, NS3, NS4A/4B, and NS5. (**B**). JEV hijacks the clathrin-mediated endocytic pathway and the clathrin-independent endocytosis pathway in epithelial cells and neuronal cells, respectively, after interaction with attachment receptors and entry receptors [15,16].

JEV is a member of the genus *Orthoflavivirus* [11] in the family *Flaviviridae* [12]. Its positive-sense single-stranded RNA genome consists of 11,000 nucleotides and encodes three structural proteins, including the enveloped protein (E), the membrane protein (M), and the capsid protein (C), as well as seven non-structural proteins named NS1, NS2A/2B, NS3, NS4A/4B, and NS5 (Figure 1) [13]. Based on the nucleotide sequence of the E gene, JEV can be further divided into five genotypes: GI-GV [1]. A detailed phylogenetic analysis of the current major global JEV strains showed that JEV originated from the same ancestor in the Indonesia–Malaysia region, and genotype I JEV has gradually replaced genotype III to become the predominant epidemic strain in the last two decades in China and even the whole of Asia [14].

The E and M proteins form heterodimers that are embedded in the viral envelope, whereas the nucleocapsid, composed of the capsid protein and the viral RNA genome, is located beneath the envelope [17]. The E protein is involved in cellular attachment and entry, and thus it is the primary target of neutralizing antibodies [18]. Meanwhile, some studies have reported that the E protein is involved in binding to the surface receptors of host cells and plays an important role in mediating virus-cell membrane fusion [19]. The role of prM in JEV entry is still largely unknown, and the lack of prM in pseudotyped viruses has no significant influence on virus entry [20]. The C protein is a highly basic protein and associates with RNA to form the nucleocapsid to mediate viral assembly and protect the viral RNA genome.

The NS proteins are responsible for viral replication and interact with host factors to modulate immune responses. NS1 is a glycoprotein that exists in both intracellular and secreted forms. NS2B and NS3 form a protease complex that processes the viral polyprotein, with NS3 acting as a bifunctional protein that harbors a proteolytic domain at its N-terminus and a helicase domain at its C-terminus. NS5 is a highly conserved bifunctional protein that consists of an MTase domain and an RdRp domain, located at the N-terminal and C-terminal respectively. Their roles in regulating immune responses will be discussed in the following sections.

### 1.3. Viral Life Cycle

JEV has a broad host tropism, and various receptors have been identified to be involved in virus attachment and entry so far, such as heparan sulfate proteoglycans, glycosaminoglycans, laminin, and other host factors, including vimentin, nucleolin, glucose-regulated protein 78 (GRP78), and the recently identified porcine entry factor CD161 [4]. Viral entry is the first step to establishing infection, and several studies have shown that the viral endocytic routes varied for different cell types. JEV hijacks the clathrin-mediated endocytic pathway and the clathrin-independent endocytosis pathway in epithelial cells and neuronal cells, respectively, after interaction with attachment receptors and entry receptors [15,16]. After internalization, virions are delivered to early endosomes and subsequently trafficked to late endosomes, where the acidic pH triggers membrane fusion and genome release into the cytoplasm for RNA synthesis [21]. Once the positive-strand RNA is released into the cytoplasm, it is translated into a polyprotein and cleaved into at least 10 proteins. The viral NS proteins (NS4A, NS4B, NS1, NS2A, NS2B, NS3, and NS5) recruit the viral genome to form a replication compartment associated with the endoplasmic reticulum (ER). The C protein forms dimers and associates with viral (+) strand RNA to bud into ER membranes containing the E-prM proteins. The immature virus particles are transported to the cell surface by the secretory pathway, where viral particles undergo a series of maturation steps, including glycosylation of prM and E, low-pH-induced rearrangement of E-prM, and prM cleavage. Mature virus particles are transported to the cell surface in vesicles and released from the cell surface by exocytosis [22] (Figure 1).

## 2. Innate Immune Response

### 2.1. Viral Sensing

**Figure 2:**
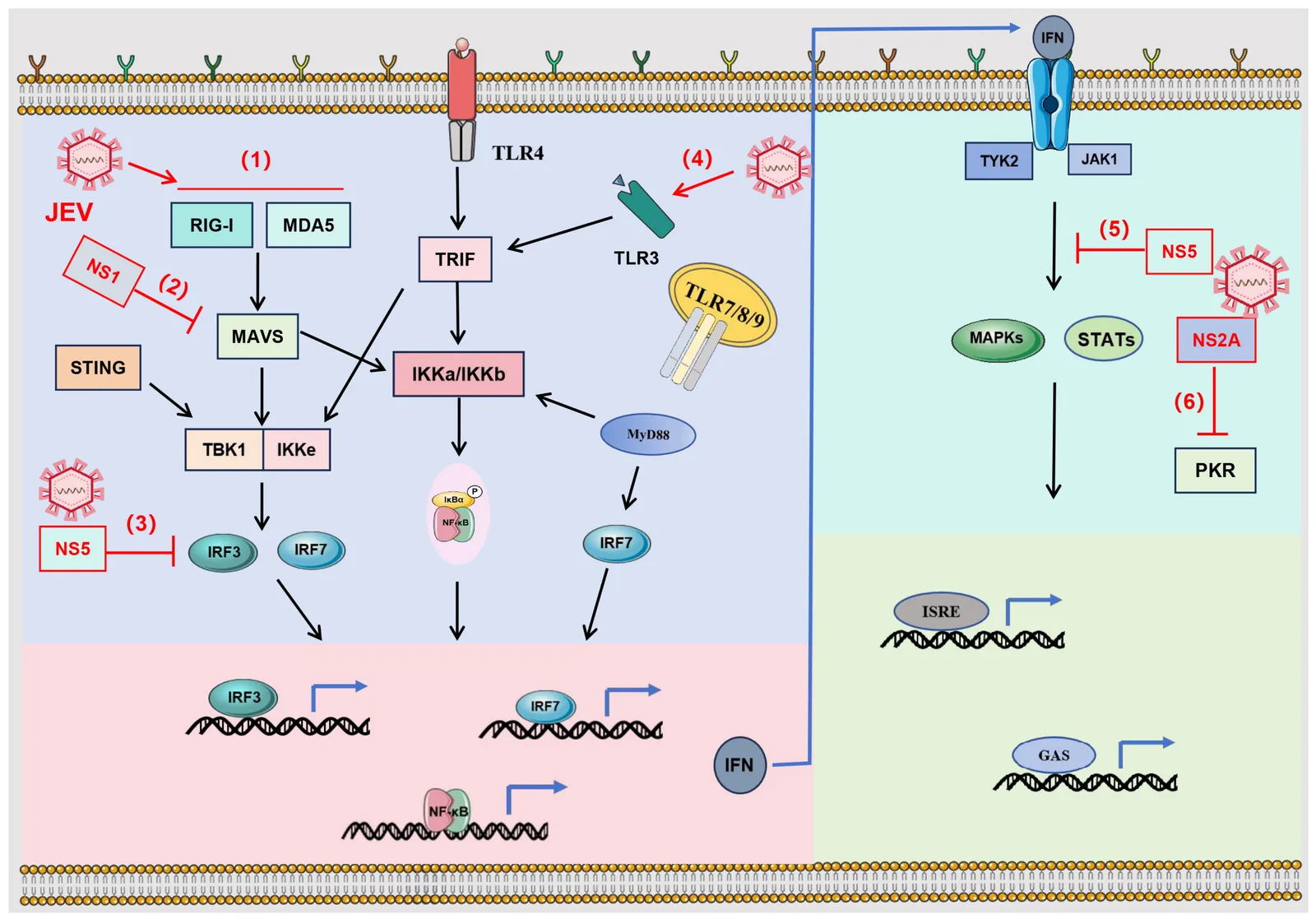
The innate immune regulation strategies of JEV. (1) JEV activated RIG-I and induced the expression of interferon (IFN) and pro-inflammatory factors [29]; (2) JEV-NS1 indirectly inhibits MAVS activation through microRNA 22 (miR-22) [30]; (3) JEV-NS5 inhibited the activation of the transcription factor IRF3 [31]; (4) JEV activated TLR3 to induce the expression of inflammatory factors [32]; (5) JEV-NS5 suppressed the JAK1-STAT signal pathways [33]; (6) JEV-NS2A inhibits protein kinase R (PKR) activation [34].

Various cell types can provide favorable environments for JEV genome replication and viral protein translation. They are recognized as foreign invaders by pattern recognition receptors (PRRs) to trigger a variety of host immune responses [23]. In mammalian cells, there are at least four major types of well-characterized PRRs, including Toll-like receptors (TLRs), Retinoic acid-inducible gene I (RIG-I)-like receptors (RLRs), cyclic GMP-AMP synthase (cGAS), and cytoplasmic NOD-like receptors (NLRs) [24,25,26,27]. PRR recognition will trigger the production of cytokines and chemokines, which induce an antiviral state. During JEV infection, the nucleic acid sensors, including RIG-I and melanoma differentiation-associated protein 5 (MDA5), along with endosomal TLR3 and TLR4, are engaged in innate immune activation (Figure 2) [28].

As viral RNA sensors, TLRs can recognize microbes in different cellular locations. TLRs 1, 2, 4, 5, and 6 are expressed on the plasma membrane to recognize various pathogen-associated molecular patterns (PAMPs) in the extracellular environment, such as bacterial lipopolysaccharide (LPS) and lipoteichoic acid. In contrast, TLRs 3, 7, 8, and 9 are mainly expressed on the endosomal and lysosomal membranes, where they detect several different nucleic acids [35]. Double-stranded RNAs (dsRNAs) are generated in the cytoplasm during the life cycle of most RNA viruses, including JEV, whose replicative intermediates are sensed by RIG-I and MDA5; single-stranded RNAs in the endosomes are likewise potent activators of the innate immune response. Studies have reported that TLR3-deficient mice had higher levels of pro-inflammatory cytokines and were extremely vulnerable to JEV by using several TLR-deficient mouse strains (TLR2, TLR3, TLR4, TLR7, TLR9) [36,37,38,39], indicating TLR3 is an antiviral factor.

TLR4 signals through both myeloid differentiation primary response 88 (MyD88) and TIR-domain-containing adapter-inducing interferon-β (TRIF) and could induce the expression of both type I IFN (IFN-I) and inflammatory genes. In addition, TLR4, located on the cell surface, has been shown to detect viral components such as envelope glycoproteins to modulate immune response [40,41]. Similarly, TLR7 has also been shown to be involved in regulating the expression of IFN-I and pro-inflammatory cytokines in response to the orthoflaviviruses Dengue virus (DENV), West Nile virus (WNV), and Zika virus (ZIKV) infection [24,36,42,43,44].

RLRs are cytosolic sensors of viral RNA that respond to viral nucleic acids, and RIG-I as well as MDA5 are two best-characterized RLRs that recognize double- and single-stranded RNA [45,46,47,48]. Notably, whereas the involvement of TLR7 in the recognition of DENV, WNV, and ZIKV is well documented, direct evidence for TLR7-mediated sensing of JEV is still limited; by contrast, the protective role of TLR3 against JEV has been established using TLR-deficient mouse models [36,37,38,39].

### 2.2. IFN-I Response

**Figure 3:**
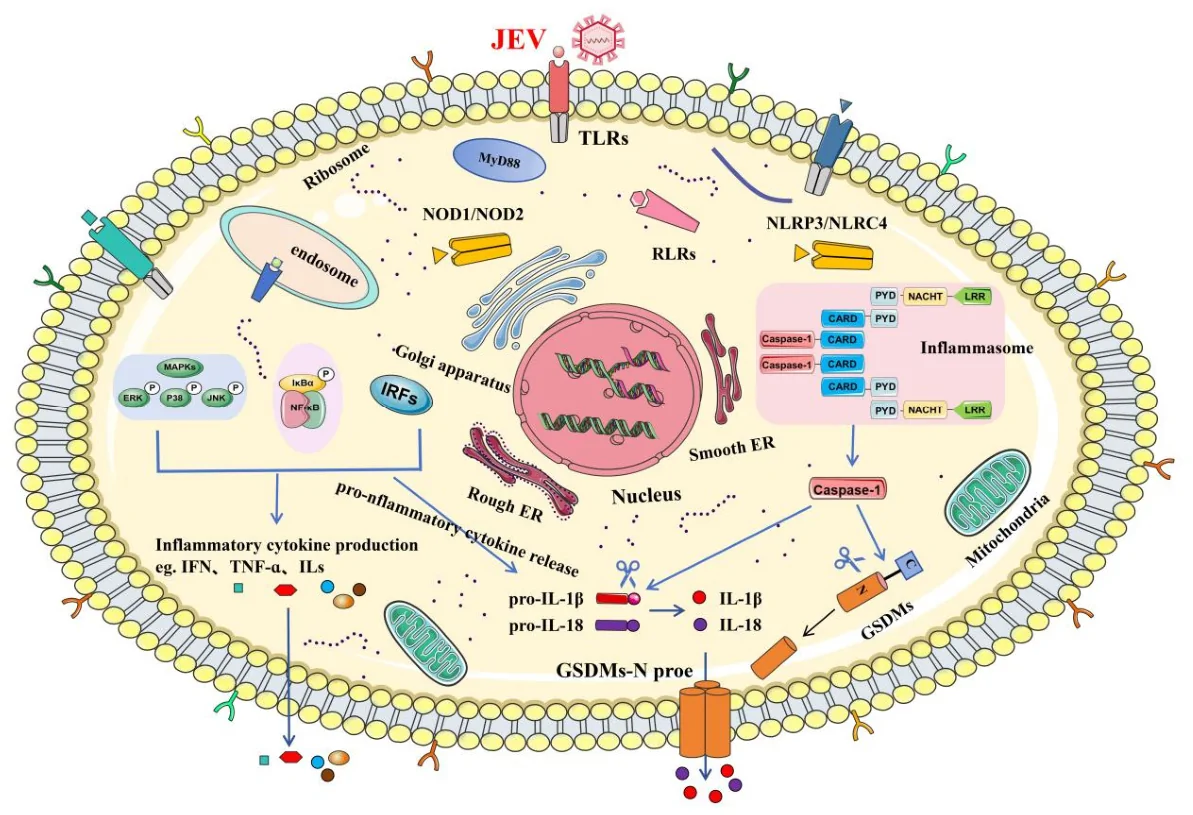
JEV infection modulates the production of IFNs and pro-inflammatory cytokines. JEV enters cells by receptor-mediated endocytosis, and viral RNA can be recognized by TLR3 and TLR4 in endosomes; viral genomes synthesized in the cytoplasm can be detected by RIG-I, cGAS, and NLRs, ultimately inducing high amounts of IFNs, pro-inflammatory cytokines, and chemokines.

The innate immune system is the first line of defense against virus propagation. During orthoflavivirus infection, viral-encoded PAMPs, including dsRNA and viral proteins, could be recognized by a variety of PRRs. PRRs serve as sentinels to detect invaders and induce the production of IFNs and inflammatory cytokines (Figure 3). JEV showed robust and prolonged viral replication in the intestine, spleen, liver, kidney, and other abdominal organs in IFN-deficient mice [49], indicating host IFN-I signaling was the major barrier to the pathogenicity of JEV. Similarly, IFN-I signal-competent mice died after a prolonged neurological illness, but IFN-I signal-incompetent mice all succumbed without neurological signs [50]. The activation of TLR3 and RLRs induces the production of IFN-I (IFN-α and IFN-β), whose most important action is to inhibit viral replication. They bind the receptors IFNAR1 or IFNAR2 to activate JAK1 and tyrosine kinase 2, leading to the phosphorylation and dimerization of STAT1 and STAT2, which then forms a complex with IRF9 to induce the expression of antiviral proteins, including protein kinase R (PKR), the 2’,5’-oligoadenylate synthetase (OAS)/RNase L system, myxovirus resistance protein 1 (Mx1), ISG15, and viperin. In addition, IFN-I can enhance the cytotoxicity of NK cells and upregulate MHC-I molecule expression to clear viral infection. Notably, IFN-γ is the principal macrophage-activating cytokine and serves critical functions in immunity against intracellular microbes. Larena M et al. reported that IFN-γ, secreted by T cells, is critical for recovery from primary JEV infection by suppressing virus growth and promoting virus clearance in the CNS [51,52].

### 2.3. Inflammatory Cytokines

One of the earliest innate immune responses to infection is the production of cytokines, which are critical for the acute inflammatory response (Figure 3). JEV pathogenicity and clinical symptoms are highly related to inflammation processes and subsequent breaches in the blood–brain barrier (BBB). However, the JEV infection-induced inflammation is a double-edged sword: on the one hand, the inflammatory response is the first line of defense against viral infection; on the other hand, the excessive or prolonged inflammatory response can cause immunologic injury instead, especially triggering the disruption of the BBB and the cell death of microglial cells [53,54]. In this section, we focus on the overall cytokine response elicited by JEV infection as part of the host innate immune defense, whereas Section 3.2 addresses how the virus actively manipulates cytokine production to evade immune clearance.

The virus completes its lifecycle in host cells and its proteins and RNAs can be detected by PRRs to trigger innate immune response and elevate the production of various kinds of interleukins. Elevated IL-6 levels are associated with disruption of BBB integrity and increased disease severity [55,56], and IL-6/IL-8 levels correlate with the severity of orthoflavivirus-associated neurological disease (detailed in Section 3.2) [57]. Meanwhile, anti-inflammatory cytokines are also induced to maintain homeostasis and prevent the inflammatory storm and inflammation-associated brain damage. For example, IL-10, as a negative immune regulator, inhibits the activation of macrophages and dendritic cells and the subsequent production of inflammatory cytokines.

### 2.4. Regulation of Host microRNAs During JEV Infection

MicroRNAs (miRNAs) are small non-coding RNAs of approximately 18–25 nucleotides that post-transcriptionally regulate gene expression, usually by binding to the 3′ untranslated regions (UTRs) of target mRNAs. Accumulating evidence indicates that host miRNAs are dynamically regulated during JEV infection and play dual roles in the virus–host interplay [58]. On the one hand, some miRNAs or miRNA-sponging transcripts exert antiviral effects. For example, a circular RNA (circRNA) network that functions as a miRNA sponge was reported to play a potential antiviral role in the early stage of JEV infection in the mouse brain [59]. On the other hand, JEV can hijack the host miRNA machinery to promote viral replication and evade innate immune responses. JEV NS1 was shown to indirectly inhibit MAVS activation through miR-22, thereby suppressing type I interferon production [30] (Figure 2). More recently, the long non-coding RNA JINR1 was reported to promote JEV infection and virus-induced cell death by regulating the miR-216b-5p/GRP78 and miR-1-3p/DDX5 axes [60], and miR-9-5p was found to regulate the transcription factor Onecut2 in JEV-infected neural stem/progenitor cells, affecting neuronal development [61]. Collectively, these findings highlight miRNAs as critical regulators of the JEV-host innate immune interaction and as promising targets for antiviral intervention [58,62].

## 3. Innate Immune Evasion

### 3.1. Inhibition and Evasion of IFN-I

The major way in which the innate immune system deals with viral infections is to induce the expression of IFN. Studies have found that JEV NS1 could inhibit the production of IFN-I by targeting MAVS [30]. JEV subgenomic flavivirus RNA (sfRNA) reduced the phosphorylation and nuclear translocation of IRF3, as well as the expression of downstream IFN-β [63]. Similarly, ZIKV sfRNA could inhibit IFN-β promoter activation mediated by RIG-I or MDA5 [64]. Interestingly, the DNA sensor cGAS-STING axis can also be effectively activated during JEV infection [65], and cGAS has also been proven to detect and limit flavivirus infection [66,67]. Recently, several new viral antagonists have been identified: JEV NS1 inhibits IFN-β production by interacting with the host RNA helicase DDX3X [68]; JEV NS4B suppresses IFN-β induction by targeting TLR3 and TRIF [69]; JEV NS1 and NS4B synergistically impair TLR3 signaling to promote viral replication [32]; and JEV NS5 degrades TRAF3 in cooperation with the host factor TUFM to suppress type I interferon induction [70].

To promote its replication, JEV has evolved multiple strategies for immune evasion to escape the innate immune responses (Figure 2). The NS5 protein, which is highly conserved among orthoflaviviruses, has been shown to regulate IFN production and signaling [33,71,72,73]: NS5 could inhibit the activation of transcription factors IRF3 and JAK-STAT to block the IFN-I signaling pathway [31,33]. In addition, JEV NS1 inhibits IFN-β production by enhancing CDK1 phosphorylation and contributes to immune evasion; JEV NS2A not only represses IFN-α production but specifically blocks PKR activation to inhibit eIF2α phosphorylation and enhances viral replication [34]. In addition, many in vitro studies have reported that JEV replication could also hamper the IFN responses by regulating ISRE and GAS-driven genes [74,75].

### 3.2. Regulation of Interleukins

In contrast to the general cytokine response described in Section 2.3, JEV actively manipulates the production and function of interleukins to subvert immune clearance and promote pathogenesis. JEV has the propensity to infect neuronal and glial cells of the brain, triggering microglial activation and the generation of pro-inflammatory cytokines [53]. Wang et al. reported that JEV infection markedly elevated IL-1α release to devastate the BBB and promoted viral neuroinvasion, indicating that IL-1α plays a crucial role in augmenting JEV-associated pathophysiology [76]. Similarly, the effect of IL-1α could be attenuated by its antagonist [76]. In addition, JEV infection could activate the inflammasome to promote the generation of IL-1β and IL-18 in microglia and astrocyte cells, which in turn differentially regulate the release of other cytokines and chemokines from these cells [77,78]. JEV infection also results in the release of a variety of chemokines and inflammatory factors, including IL-6, which plays a pivotal role in the enhancement of endothelial permeability and may be related to endothelial barrier damage [51,79,80]. In addition, the elevated levels of pro-inflammatory cytokines and the altered permeability of the BBB may be positively related. The high amount of IL-6 can digest BBB tight junctions, leading to CNS inflammation [55,56]. Another similar study reported that in patients with neurological diseases caused by orthoflavivirus infection, the production of IL-6 and IL-8 increased with the severity of JEV infection [57].

Notably, there are also anti-inflammatory cytokines, such as IL-4 and IL-10, to prevent the inflammatory storm and inflammation-associated brain damage during JEV infection. These anti-inflammatory cytokines are negatively associated with neuronal death, and the expression of both was reduced during the progression of JEV infection [81]. IL-10 acts as an immune regulator to protect tissues from damage caused by over-adaptive immune responses and pro-inflammatory mediators. A reduction in JEV-mediated IL-10 generation may be associated with microglial activation and neuronal death [82,83].

### 3.3. Other Immune Evasion Mechanisms

**Figure 4:**
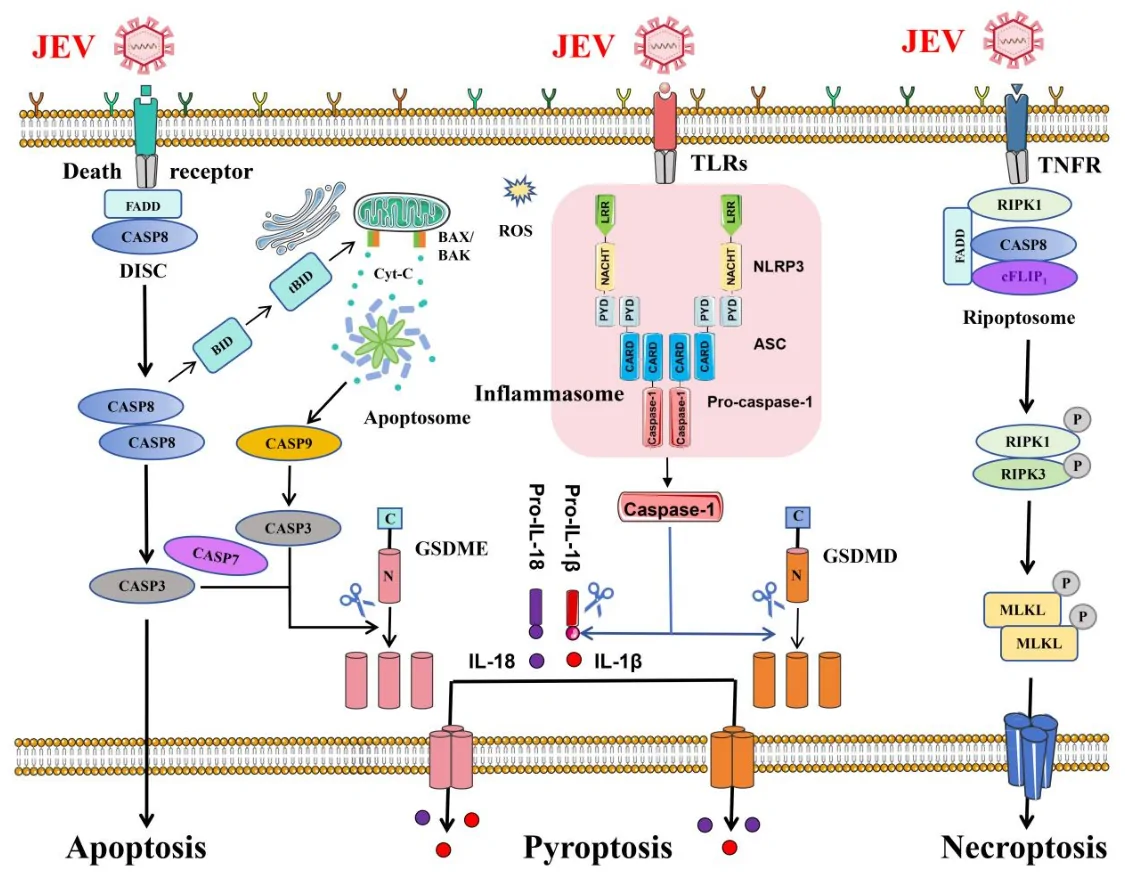
Programmed cell death pathways during JEV infection. Apoptosis: JEV infection initiates a signal cascade mediated by caspase-8 and caspase-3 to initiate inflammatory cell death. Pyroptosis: The viral proteins of JEV act as cytoplasmic PAMPs or TLRs to induce inflammasome assembly and stimulate caspase-1 activation, initiating pyroptosis. Necroptosis: JEV infection triggers necroptosis, which depends on the formation of RIPK1 and RIPK3 complexes and the activation of downstream MLKL proteins to form channels in the membrane.

Programmed cell death, although an important host defense, can be subverted by many intracellular pathogens to favor viral dissemination. In the context of JEV infection, the virus can cross the BBB and efficiently replicate in astrocytes and microglia, leading to neuronal death through multiple mechanisms [53]. In this process, a variety of pro-inflammatory cytokines drive cells to undergo apoptosis, pyroptosis, and necroptosis (Figure 4) [84].

Apoptosis is a major kind of cell death and driven by the initiator caspase-8, -9, and -10, as well as the cleavage executor caspase-3 and -7. Among them, caspase-3 and -8 can cleave and activate gasdermin E (GSDME) or GSDMD, respectively, thereby leading to inflammatory cell death [85,86]. It is reported that apoptosis always occurs during JEV infection in neuronal and non-neuronal cells through multiple signal pathways [87]. There are three extensively studied apoptosis pathways according to their initiator caspases [88]. On the one hand, JEV infection could induce mitochondrial-mediated apoptosis through the proapoptotic protein BAX [89] and also induce apoptosis by the IRE1/JNK pathway of ER stress [90]. On the other hand, JEV inhibits the STAT3-Foxo-Bcl-6/p21 pathway to trigger apoptosis [91]. Regarding viral proteins, NS3 of JEV induces cell apoptosis by activating caspase-3 or caspase-8, causing extensive damage [92,93]; the M protein of JEV, WNV, and Yellow fever virus (YFV) also has pro-apoptotic properties [94,95]. In addition, JEV-NS4B could also activate the PERK-ATF4-CHOP pathway to lead to apoptosis in response to ER stress [96]. Furthermore, to promote its replication, JEV infection activates PI3K signaling to block caspase-dependent apoptosis at the early stage of virus infection [97].

Pyroptosis is another form of cell death induced by pro-inflammatory cytokines in microglia during JEV infection [98]. JEV infection could activate the Src/Ras/Raf/ERK/NF-κB signaling axis in the neuron/glial co-culture system in a ROS-dependent manner [99]. Meanwhile, when cells were treated with inhibitors targeting PTK, Ras, and ERK pathways, the production of JEV-induced pro-inflammatory cytokines was significantly inhibited, similar to alleviating neurotoxicity [99]. The activation of neurotoxic microglia and subsequent inflammatory responses are strongly correlated with JEV-induced CCR2 expression [100]. Furthermore, ROS and K+ efflux induce NLRP3 inflammasome assembly in JEV-infected mouse microglia and brain tissue, leading to caspase-1 activation and cytokine maturation [77].

Necroptosis is a regulated form of necrotic cell death mediated by the kinase activities of receptor-interacting protein kinase 1 (RIPK1) and RIPK3, which phosphorylate mixed lineage kinase domain-like protein (MLKL), leading to MLKL oligomerization and plasma membrane pore formation [101]. During JEV infection, transcriptomic analysis of JEV-infected macrophages revealed activation of the necroptotic pathway, which was confirmed by immunofluorescent staining with specific markers [102]. In addition, necroptosis is associated with neuronal loss during JEV infection, providing evidence that necroptosis participates in the pathogenesis of JEV [103].

## 4. Future Directions and Therapeutic Prospects

### 4.1. Unresolved Questions and Knowledge Gaps

Despite substantial progress in understanding JEV-host innate immune interactions, several key questions remain unresolved. First, although multiple attachment and entry factors have been identified in vitro, the physiologically relevant receptor(s) mediating JEV entry in vivo remain to be defined [104]. Second, the precise role of the cGAS-STING axis in JEV sensing and pathogenesis warrants further investigation, as both antiviral and pro-inflammatory activities have been proposed [65,66,67]. Third, the in vivo functions of most JEV-regulated host microRNAs and long non-coding RNAs remain largely unexplored, and gain- or loss-of-function studies in animal models are needed to establish their physiological relevance [58,59,60,61]. Finally, the molecular mechanisms by which JEV breaches the BBB and the relative contributions of neuroinflammation versus direct viral cytopathology to neurological sequelae are still incompletely understood [80].

### 4.2. Developments in Emergency Antiviral Therapy

Because JE progresses rapidly and no specific antiviral drug is currently licensed, there is an urgent need for effective emergency therapies [105]. Several promising directions are under active investigation. Small-molecule inhibitors that target viral enzymes or host factors essential for viral entry and replication are being developed; for example, bergamottin, a bioactive component of bergamot, was recently reported to dually inhibit JEV internalization and genome replication in vitro [106]. Host-directed strategies, such as targeting the entry factor CD161, have also been proposed to inhibit JEV infection [107]. In addition, RNA interference (RNAi)-based therapeutics, including siRNAs and miRNAs directed against conserved viral genomic regions, represent a rapidly advancing modality with multiple delivery platforms under evaluation [62]. Given that antibodies cannot readily access the CNS, anti-inflammatory and neuroprotective approaches that temper the excessive inflammatory response while preserving antiviral immunity may complement direct-acting antivirals and help reduce mortality and neurological sequelae [105].

### 4.3. Future Research Directions

Beyond immediate therapeutic goals, several research avenues deserve priority. From a mechanistic perspective, systematic dissection of the JEV-encoded proteins that antagonize innate immune signaling (e.g., NS1, NS2A, NS4B, and NS5) continues to reveal new immune-evasion nodes, as exemplified by recent findings on NS1-DDX3X [68], NS4B-TLR3/TRIF [69], and NS5-mediated TRAF3 degradation [70]. The application of high-throughput technologies, including single-cell and spatial transcriptomics, will help define cell-type-specific innate immune responses during JEV neuroinfection. Our group has recently demonstrated that JEV NS1 evades the type I interferon response by interacting with the host helicase DDX3X [68], and we are currently constructing recombinant JEV mutants and using reverse genetics approaches to map the viral determinants of innate immune evasion and neurovirulence. We anticipate that integrating these mechanistic insights with vaccine and therapeutic development will ultimately reduce the burden of this devastating disease.

In summary, the interplay between JEV and the host innate immune system is a dynamic battlefield in which the virus employs multiple strategies to escape recognition and antagonize antiviral effectors, while the host mounts multilayered innate defenses. A comprehensive understanding of these mechanisms not only deepens our insight into viral pathogenesis but also provides a rational basis for the development of effective vaccines, immunomodulatory interventions, and antiviral therapies for Japanese encephalitis.

## Data Availability

No new data were created or analyzed in this study. Data sharing is not applicable to this article.
